# Effects of oxytocin on prosocial behavior and the associated profiles of oxytocinergic and corticotropin-releasing hormone receptors in a rodent model of posttraumatic stress disorder

**DOI:** 10.1186/s12929-019-0514-0

**Published:** 2019-03-21

**Authors:** Sheng-Chiang Wang, Chen-Cheng Lin, Nian-Sheng Tzeng, Che-Se Tung, Yia-Ping Liu

**Affiliations:** 1 Graduate Institute of Medical Sciences, National Defense Medical Center, No. 161, Section 6, Minquan East Road, Neihu District, Taipei City, 114 Taiwan; 2 Department of Psychiatry, Tri-Service General Hospital Songshan Branch, No. 131, Jiankang Road, Songshan District, Taipei City, 105 Taiwan; 3 Department of Psychiatry, Cheng Hsin General Hospital, No. 45, Chenghsin Street, Beitou District, Taipei City, 112 Taiwan; 4 Department of Psychiatry, Tri-Service General Hospital, School of Medicine, National Defense Medical Center, No. 325, Section 2, Chenggong Road, Neihu Distict, Taipei City, 114 Taiwan; 5 Student Counseling Center, National Defense Medical Center, No. 161, Section 6, Minquan East Road, Neihu District, Taipei, 114 Taiwan; 6 Laboratory of Cognitive Neuroscience, Department of Physiology and Biophysics, National Defense Medical Center, No. 161, Section 6, Minquan East Road, Neihu District, Taipei City, 114 Taiwan; 7 Division of Medical Research & Education, Cheng Hsin General Hospital, No. 45, Chenghsin Street, Beitou District, Taipei City, 112 Taiwan, Republic of China

**Keywords:** Corticotropin-releasing hormone, Fear circuit areas, Oxytocin, Posttraumatic stress disorder, Prosocial behavior, Single prolonged stress.

## Abstract

**Background:**

Traumatic experience may lead to various psychological sequelae including the unforgettable trauma-associated memory as seen in posttraumatic stress disorder (PTSD), with a mechanism of impaired fear extinction due to biological imbalance among hypothalamic-pituitary-adrenal (HPA) axis and fear circuit areas such as medial prefrontal cortex (mPFC), hippocampus, and amygdala. Recently the impaired sociability seen in PTSD patients received great attention and the involvement of oxytocin (OXT) mediation is worth being investigated. This study examined whether the trauma-altered prosocial behavior can be modulated by OXT manipulation and its relationship with corticotropin-releasing hormone (CRH) signaling.

**Methods:**

Male rats previously exposed to a single prolonged stress (SPS) were evaluated for their performance in social choice test (SCT) and novel object recognition test (NORT) following the introduction of intranasal oxytocin (OXT) and OXT receptor antagonist atosiban (ASB). OXT receptors (OXTR) and CRH receptors (CRHR1, CRHR2) were quantified in both protein and mRNA levels in medial prefrontal cortex (mPFC), hippocampus, and amygdala.

**Results:**

SPS reduced inclination of rats staying at the sociable place with performing less prosocial contacts. OXT can amend the deficit but this effect was blocked by ASB. Expression of OXTR became reduced following SPS in mPFC and amygdala, the latter exhibited higher therapeutic specificity to OXT. Expression of CRHR1 appeared more sensitive than CRHR2 to SPS, higher CRHR1 protein levels were found in mPFC and amygdala.

**Conclusion:**

Psychological trauma-impaired sociability is highly associated with OXT signaling pathway. Intranasal OXT restored both the SPS-impaired prosocial contacts and the SPS-reduced OXTR expressions in mPFC and amygdala. OXT may have therapeutic potential to treat PTSD patients with impaired social behaviors.

## Introduction

People experienced psychological trauma may suffer from many sequelae such as hyperarousal, depressed mood, emotional avoidance, and preoccupation of event-related intrusive/unforgettable fear memory as described by the symptoms of posttraumatic stress disorder (PTSD) [1]. Increasing evidence reported that traumatic experiences are also known to cause poor interpersonal sociability [2–4], consistent with the impairment of interpersonal relationship in PTSD patients, and that perceived social support may serve as a buffer against psychological distress and therefore reduce the risk of developing PTSD [5]. Since the underlying mechanism of PTSD remains unsolved, the efficacy of pharmacotherapy in treating PTSD-associated mental problems has not claimed compelling efficacy [6]. For example, SSRIs failed to correct the non-adherence to cognitive psychotherapy in PTSD patients [7, 8], and also failed in restoring psychological trauma-induced fear extinction abnormalities in a rat model of PTSD [9]. It is worth investigating whether an adjunct therapeutic agent could be employed in this regard. Attention has focused on oxytocin (OXT) as it is involved in both stress-related sociability and stress-induced dysfunction of hypothalamic-pituitary-adrenal (HPA) axis [10, 11].

The OXT profile is highly relevant to HPA dysfunction following traumatic stress. OXT may inhibit the action of corticotropin-releasing hormone (CRH), which induces the release of cortisol during stress response [12]. Central OXT may modulate the social and cognitive effects of stress [13–15]. Given that social interaction is beneficial in reducing stress response with possibly affecting the plasticity and stability of brain epigenetic process [16], OXT-promoted social activity may play a key role in managing stress by driving social responses to divergent stress [17–19], in line with the rodent evidence that OXT receptors (OXTR) were found important in regulating social recognition, interaction, and reward [20]. Additionally, central OXT signaling is heavily involved in the buffering effect of social relationships against stressful events and experiences, thereby reducing vulnerability to physiological and behavioral consequences [21]. Furthermore, intranasal oxytocin raises peripheral levels of oxytocin [22, 23], and may cross the blood brain barrier with biological relevance [3]. Moreover, intranasal oxytocin can be delivered to peripheral circulation and lead to afferent feedback to the brain from peripheral organs rich in oxytocin receptors [24]. Evidence has indicated that early and repeated administration of intranasal OXT following trauma may not only prevent the development of PTSD symptoms but also improve prosocial behaviors [25, 26]. However, so far no study has directly examined the role of brain region-dependent OXTR in prosocial behaviors following psychological traumatic stress.

In terms of stress-induced hormonal alterations, OXT signaling pathway is mediated by rapid feedback inhibition of corticosteroids [27, 28], with primarily associated with CRH effects [29]. CRH is a neuropeptide released by the hypothalamus after experiencing stress, and plays a key role in modulating physiological and behavioral response to stressors [30]. Along with the hypothalamic paraventricular nucleus, large amounts of CRH are also present in the amygdala, modulates various autonomic activities and sociability under exposure to stress and fear [31, 32]. Two CRH receptors (CRHRs), CRHR1 and CRHR2 are also involved in the modulation of stress response, with possibly different biological roles, i.e., anxiogenic effects of CRHR1 and anxiolytic effects of CRHR2 [30, 33, 34]. Further evidence suggested that CRHRs exert their stress-modulating effects after exposure to trauma via a brain-region dependent manner [35], along with the findings that CRH activity in the medial prefrontal cortex (mPFC) increased stress-induced HPA activity and anxiety-related behaviors [36]. Therefore, detailed descriptions of OXT-CRH mediations of trauma-altered social behaviors over fear circuit areas are worth being investigated.

In the present study, we examined the hypothesis that traumatic stress–impaired sociability is associated with the OXT signaling pathway. Specifically, rats experienced single prolonged stress (SPS) may engage in a social choice test (SCT) to present their prosocial behaviors toward a rat restrained in a similar circumstance as the tested rats did. An SPS paradigm was employed because it is useful in examining the neurobiological mechanism of social impairment after traumatic stress [37, 38]. Novel object recognition test (NORT), a useful cognitive tool to examine the tendency of rats to explore a novel or unfamiliar object [39], was also employed in the present study to assess the possibility that the prosocial behavior may be confounded by curiosity. We further investigated whether OXT may enact a therapeutic potential to reverse the trauma-impaired prosocial behavior by pharmacological interventions of OXT and OXT antagonist. Finally, OXT receptors (OXTR) and CRH receptors (CRHR1, CRHR2) were quantified in both protein and mRNA levels in (mPFC), hippocampus, and amygdala to present detailed descriptions of OXT-CRH mediations of trauma-altered social behaviors over fear circuit areas. The results of the present study not only demonstrate a practical paradigm to approach how previous fear experience affects individual’s prosocial inclination, but also provide information to support the potential use of OXT in treating PTSD-impaired sociability.

## Materials and methods

### Animals and PTSD model

A total of 30 male Sprague–Dawley rats (BioLASCO Taiwan Co., Ltd.) were used. The rats were aged 8 weeks and had been weaned upon arrival at the animal center of the National Defense Medical Center (Taipei, Taiwan; Republic of China). They were housed in groups of three in a temperature- and humidity-controlled holding facility with 12-h light–dark cycles (lights on from 07:00 to 19:00). Food (standard laboratory chow diet; Ralston Purina, St. Louis, MO, USA) and sterile water were available ad libitum. Rats were randomly assigned to SPS (*n* = 18, including six for comparison of OXT receptor and its antagonist) or control (CON, *n* = 12) groups. The SPS procedures [40, 41] consisted of the following steps. First, the rats were sequentially restrained in a plastic cone for 2 h and then forced to swim in a tank of water (22-in. diameter, 20 °C) for 20 min. Following a 15-min recuperation period, they were exposed to diethyl ethyl vapor (Sigma, St. Louis, MO, USA) until they became anesthetized and unresponsive. They were then immediately returned to their home cages and left in isolation for 7 days, thus making them susceptible to impaired extinction of the fear response. During the SPS procedure, the CON group rats remained in their home cages. All of the behavioral tests were conducted between 08:00 and 18:00, and all of the rats were tested at the same time every day when possible. The experimental procedures and ethics were approved by the National Defense Medical Center’s animal care committee, and all efforts were made to reduce the number of animals used and minimize their suffering during the experiments.

### Experimental design

**Fig. 1:**
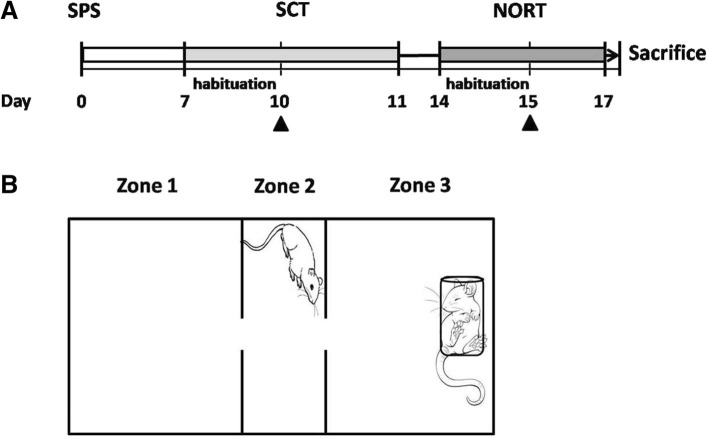
Flow chart of experimental procedures (**a**) and design of the social choice test (**b**). Arrowheads refer to the point of drug intervention, and drugs are also given during habituation periods. SPS: single prolonged stress; SCT: social choice test; NORT: novel object recognition test

Rats in SPS or CON conditions were subjected to a pharmacological regime before each behavioral test and 3-day (SCT)/ 1-day (NORT) periods of habituation to intranasal administration. Synthetic OXT (OXT 1 μg/μL, 2 × 10 μL; cat. no.: O4375, Sigma-Aldrich) was administered intranasally; dosages for OXT were chosen based on previous and preliminary studies [42–44], and the OXT receptor antagonist atosiban (ASB, cat. no.: A3480, Sigma-Aldrich) was administered intraperitoneally (5 mg/kg) 30 min prior to delivery of the OXT [45]. Saline vehicle (VEH) was also intranasally administered to the rats not receiving OXT. The subgroups were thus as follows: CON-VEH, CON-OXT, SPS-VEH, SPS-OXT, and SPS-ASB/OXT (*N* = 6 in each). We sequentially administered two behavioral tasks to evaluate all groups, namely the social choice test (SCT) at day 10, and novel object recognition test (NORT) at day 15. Neurochemical data (OXTR, CRH, CRHR1 and CRHR2 of stress-related regions) were obtained to measure the central effects of experiencing SPS. Moreover, we observed dynamic changes in the biochemical and behavioral aspects of the SPS rats under the pharmacological interventions (Fig. 1a).

### Social choice test

We used a social interaction task to evaluate the rats’ social behavior. The apparatus consisted of three chambers: the nonsocial zone (zone 1), the center (zone 2), and the social zone (zone 3). Each group of rats was tested using the SCT (Fig. 1b) to assess their social responses and empathy-like behaviors [38, 46]. Each SCT trial lasted 300 s, with a conspecific non-familiar rat in a plexiglass rodent restrainer (25 × 8.75 × 7.5 cm^3^, Harvard Apparatus, Holliston, MA, USA) placed in zone 3. The restrained rat could move and turn inside the restrainer, which had several small lateral slits and one large slit at the rear, enabling tactile and olfactory communication between the rats. The restrained rat was matched in sex and size to the tested rat. All of the tested rats were habituated to the three-chamber box (nontransparent white acrylic box; 60 × 30 × 30 cm^3^) for 10 min a day during the initial 3 days. The rats also received an intranasal drip of normal saline during the subsequent 2 days to reduce bias. On day 10, 3 days after habituation, we placed the tested rats in zone 2. Interactions between the tested and restrained rat and time spent in zone 1, zone 3, and the center (zone 2) were recorded by an overhead video recorder (TopScan, Clever Sys., Inc., VA, USA). A rat was counted as being in a zone only when all four of its paws were in that zone. All behavior was recorded and scored by two experimenters who were blind to the treatment conditions. The total time spent in each zone and the frequency and duration of contact were calculated and analyzed to identify any preference to interact with or avoid the restrained rat.

### Novel object recognition test

The NORT used in this study was modified from that of Eagle et al. [38] to exclude curiosity effects under SPS and reduce bias regarding object recognition, such as testing time, different trials, and dissimilar objects**.** The test was performed in the nontransparent open area of a black acrylic box (60 × 60 × 30 cm^3^) using the same overhead video recorder. After a 30-min habituation session on the first day, on day 2 each rat was allowed to explore two identical objects (white cylinders, diameter = 8 cm and height = 19 cm) for a familiarization time sufficient to complete 10 min of exploration of each object. On day 3, one of the objects previously used was replaced by a novel object (a five-colored rectangular prism, 6 × 6 × 17 cm^3^). The rats were placed in the open area again for 3 min, and the duration of exploration of each object (defined as sitting by, sniffing, or touching the objects) was recorded. A recognition index was calculated by dividing the time of novel object exploration by the total exploration time (novel plus familiar objects), multiplied by 100 [47].

### Analysis of mRNA expression

The rats were sacrificed through decapitation, and brain tissues containing the hippocampus, mPFC, and amygdala were rapidly dissected and immediately frozen at − 70 °C. Tissues were homogenized in the lysis buffer of a MagNA Pure Compact RNA Isolation Kit using MagNA Lyser (Roche Molecular Diagnostic, Mannheim, Germany). Total RNA was extracted using the MagNA Pure Compact System (Roche). The quantity of RNA was determined using a NanoDrop One spectrophotometer (Thermo Scientific), and the DNA sequence was evaluated using Primer Express software. The primers were synthesized by Mission Biotech Ltd. (Taipei, Taiwan). Quantitative real-time polymerase chain reaction (qPCR) was used to analyze *OXTR*, *CRH*, *CRHR1*, and *CRHR2*. Sequences for the specific primer sets used in qPCR were presented for each gene, as follows: *CRH* (F: CCACCTTCTGAGGGAAGTCTTG, R: CAACATTTCATTTCCCGATAATCTC), *CRHR1* (F: TGGTGGCCTTTGTCCTCTTC, R: GTGGCGTTGCGTAGGATGA), *CRHR2* (F: TGTTTGTGGAAGGCTGCTACCT, R: GGTATGCACCATCCAATGAAGA), and *OXTR* (F: GCTGCAACCCGTGGATCTAC, R: CGGCTGCCCTTCAGGTAAC). *GAPDH* (F: GGTGGACCTCATGGCCTACA, R: CAGCAACTGAGGGCCTCTCT) was used as a housekeeping gene. RNA samples were reverse-transcribed for 120 min at 37 °C using a High Capacity cDNA Reverse Transcription Kit (Applied Biosystems), according to the supplier’s standard protocol. cDNA derived from 10 ng of RNA was used for qPCR under the following conditions: 10 min at 95 °C, 40 cycles of 15 s at 95 °C, and 1 min at 60 °C using a 2× Power SYBR Green PCR Master Mix (Applied Biosystems) and 200 nM forward and reverse primers. Each assay was performed in triplicate on an Applied Biosystems 7900HT Real-Time PCR system, and expression fold changes were derived using the comparative C_T_ method, with *GAPDH* as an endogenous control and the CON/VEH sample as a calibrator.

### Data and statistical analysis

All statistical analyses were performed using SPSS (version 16.0, SPSS, Inc., Chicago, IL, USA) and SigmaPlot (version 12.1, Systat Software Inc., San Jose, CA, USA). Multiple types of analysis of variance (ANOVA) were performed to compare among more than two groups (with SPS and DRUG as independent variables). The significant main effects were then analyzed in post hoc comparisons using the Tukey method or Student’s t-test. A threshold of *p* < 0.05 was considered statistically significant.

## Results

### Mitigation of SPS-induced social deficits through OXT

**Fig. 2:**
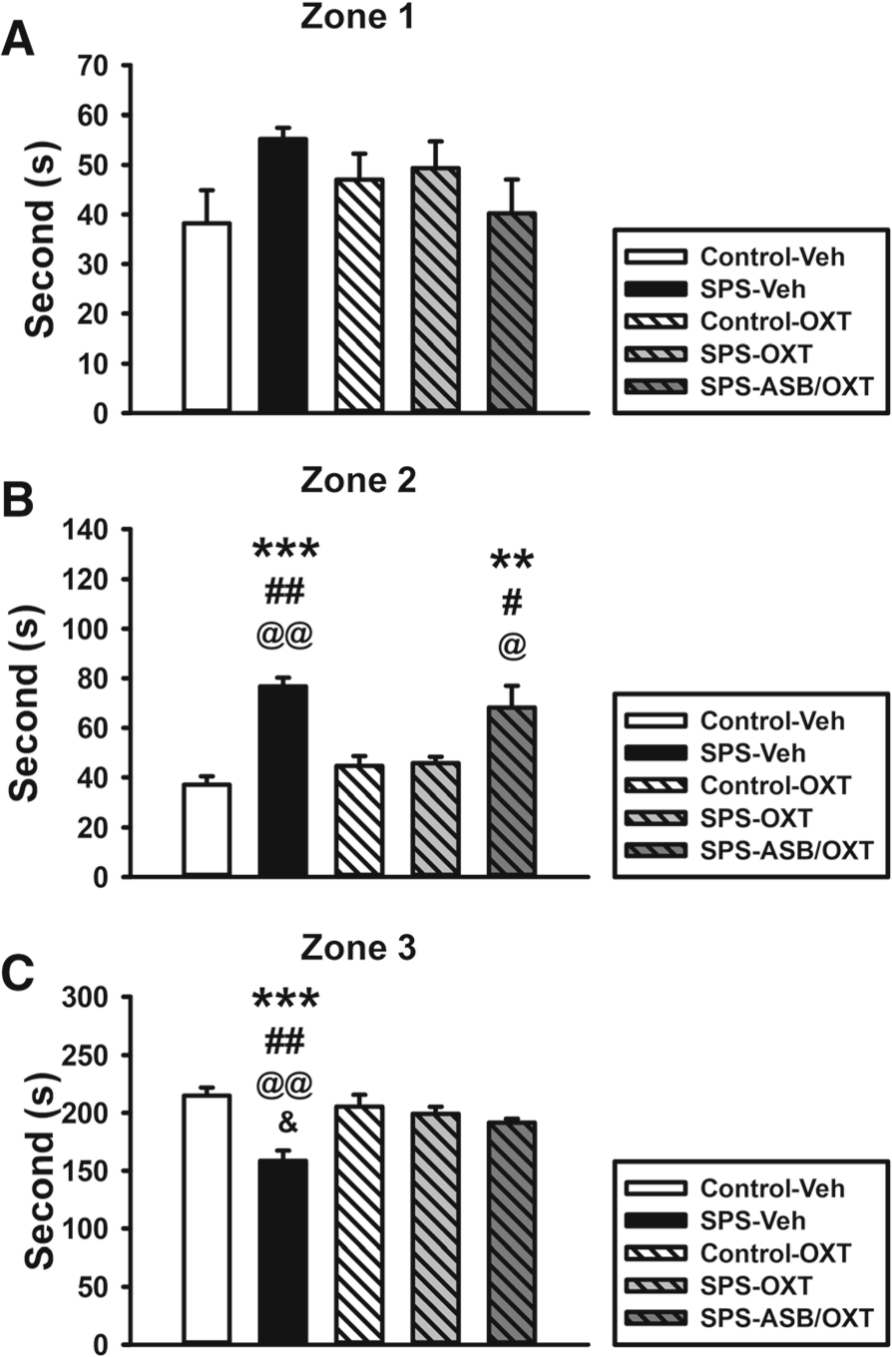
The staying Time of rats in the zone 1 (**a**), zone 2 (**b**), and zone 3 (**c**) of social choice test (SCT). SPS and drugs (OXT and ASB) did not affect the staying time in zone 1 (**a**). OXT reversed the SPS-increased of staying time in zone 2, but this could also be blocked by ASB (**b**). OXT reversed the SPS-induced decrease of staying time in zone 3, but this could be blocked by ASB (C). SPS = single prolonged stress, VEH = vehicle, OXT = oxytocin, ASB = atosiban. Bars represent mean ± SEM, **p < 0.01, ***p < 0.001, compared with Control-Veh; #*p* < 0.05, ##*p* < 0.01, compared with Control-OXT; ^@^*p* < 0.05, ^@@^*p* < 0.01, compared with SPS-OXT; ^&^*p* < 0.05, compared with SPS-ASB/OXT. *N* = 6

We subjected the rats to the SCT to determine whether SPS procedure could reduce prosocial behaviors and OXT treatment would mitigate this consequence. For the time of staying in the zone 1 (non-social zone), one-way ANOVA revealed no significant difference among all groups (F_(4, 25)_ = 1.567, *p* = 0.214) (Fig. 2a). Due to the possible confounding effect of anxiety, the time of stay in zone 2 was analyzed; ANOVA indicated a significant difference among the groups (F_(4, 25)_ = 11.744, *p* < 0.001), further analysis exhibited that the SPS-Veh rats spent more time in the zone 2 than the CON-Veh (*p* < 0.001), CON-OXT (*p* = 0.001), and SPS-OXT rats (*p* = 0.002) rats. Besides, SPS-ASB/OXT rats also spent more time in the zone 2 than the CON-Veh (*p* = 0.002), CON-OXT (*p* = 0.02), and SPS-OXT rats (*p* = 0.028) rats (Fig. 2b). For the time of staying in the zone 3 (social zone), one-way ANOVA revealed a significant difference among all groups (F_(4, 25)_ = 8.211, *p* < 0.001), deriving from the less time of staying in the zone 3 in the SPS-Veh group compared with the CON-Veh (*p* < 0.001), CON-OXT (*p* = 0.002), SPS-OXT (*p* = 0.007), and SPS-ASB/OXT groups (*p* = 0.034) (Fig. 2c).

**Fig. 3:**
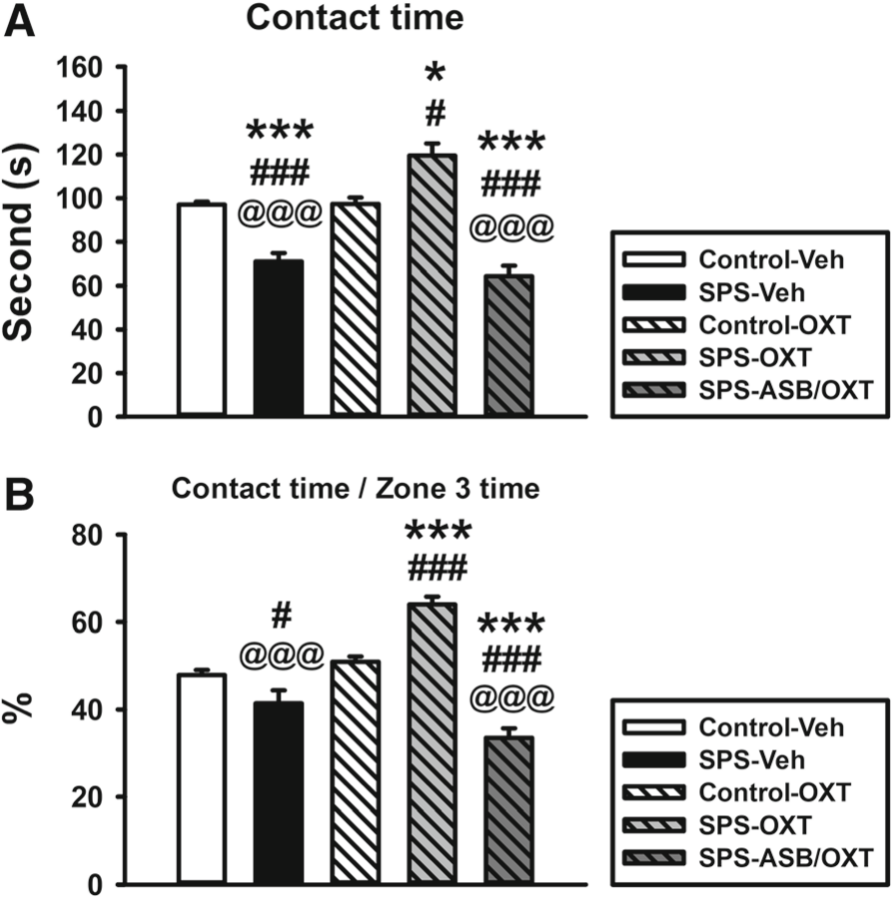
The social contacting time (**a**) and its percentage in zone 3 (**b**) of social choice test (SCT). OXT reversed SPS-induced attenuation of contact time with the restrained rat, but this could be blocked by ASB (A-B). SPS = single prolonged stress, VEH = vehicle, OXT = oxytocin, ASB = atosiban. Bars represent mean ± SEM, *p < 0.05, ***p < 0.001, compared with Control-Veh; #*p* < 0.05, ###*p* < 0.001, compared with Control-OXT; ^@^*p* < 0.05, ^@@@^*p* < 0.001, compared with SPS-OXT. N = 6

For the contact time with a social partner, a significant difference existed among the groups (F_(4, 25)_ = 31.843, *p* < 0.001); further analysis showed that SPS-Veh rats had less contact time than the CON-Veh (*p* < 0.001), CON-OXT (*p* < 0.001), and SPS-OXT rats (*p* < 0.001). Moreover, SPS-ASB/OXT rats also showed less contact time than the CON-Veh (*p* < 0.001), CON-OXT (*p* < 0.001), and SPS-OXT rats (*p* < 0.001). In addition, SPS-OXT rats displayed more contact time than the CON-Veh (*p* = 0.004) and CON-OXT rats (*p* = 0.005) (Fig. 3a). When using percentages to compare the time period of social contacts in the zone 3, a significant difference also existed among all groups (F_(4, 25)_ = 33.059, *p* < 0.001), and further analysis exhibited that the higher percentage of social contacting time in SPS-OXT group than the other groups (*p* < 0.001). Furthermore, SPS-Veh group showed lower percentage than the CON-OXT group (*p* = 0.018), and SPS-ASB/OXT group also showed lower percentage than the CON-Veh (*p* < 0.001) and CON-OXT groups (*p* < 0.001) (Fig. 3b).

**Fig. 4:**
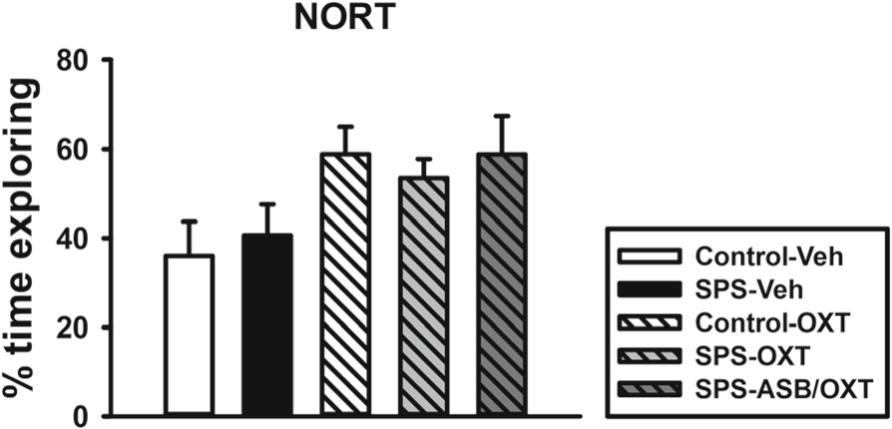
The novel object recognition test (NORT). SPS and drugs (OXT and ASB) did not affect the time percentage for recognizing a novel object. SPS = single prolonged stress, VEH = vehicle, OXT = oxytocin, ASB = atosiban. Bars represent mean ± SEM. N = 6

We further subjected the rats to the behavioral test of NORT to ensure that the curiosity is neither a confounding nor obscuring variable toward the prosocial behavior. One-way ANOVA revealed no significant difference in NORT among all of the groups (F_(4, 25)_ = 2.352, *p* = 0.082). The results indicated the prosocial behavior of rats was not affected by their curiosity (Fig. 4).

### The mRNA and protein changes of OXTR, CRHR1, CRHR2, and CRH in mPFC, hippocampus, and amygdala

**Fig. 5:**
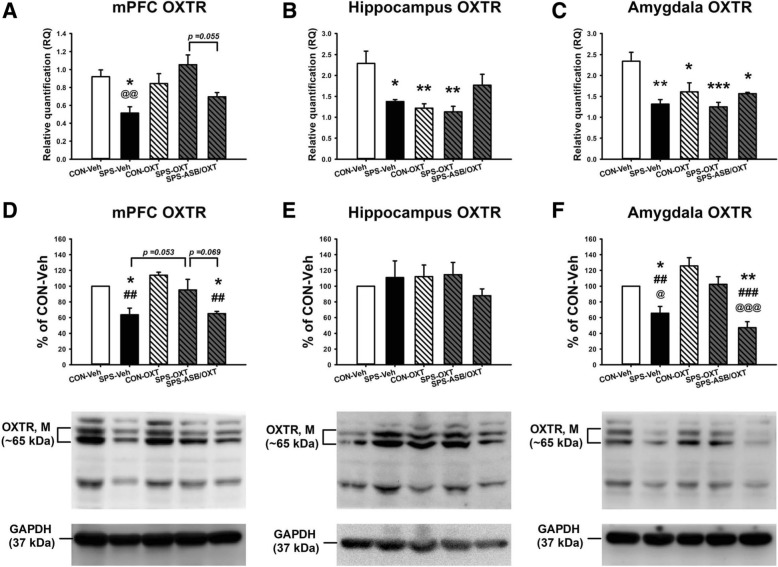
The OXTR mRNA levels and protein expressions in the mPFC, hippocampus, and amygdala. SPS reduced the OXT mRNA level in the mPFC, hippocampus, and amygdala, but only the mPFC OXTR mRNA level can be reversed by OXT administration (**a**-**c**). OXT reversed the SPS-decreased OXT protein expressions in the mPFC and amygdala, and these effects can be blocked by ASB (D, F). SPS and drugs (OXT and ASB) did not affect the hippocampus OXT protein expression (E). SPS = single prolonged stress, VEH = vehicle, OXT = oxytocin, ASB = atosiban, OXTR = oxytocin receptor. Bars represent mean ± SEM, *p < 0.05, **p < 0.01, ***p < 0.001, compared with Control-Veh; ##*p* < 0.01, ###*p* < 0.001, compared with Control-OXT; ^@^*p* < 0.05, ^@@^*p* < 0.01, ^@@@^*p* < 0.001, compared with SPS-OXT. *N* = 5 for the mRNA levels (A-C); *N* = 4 for the protein expressions (**d**-**f**)

After the above behavioral experiments, the rats were sacrificed and their brains were removed to measure the OXTR, CRHR1, CRHR2 mRNA and protein levels and CRH mRNA levels in mPFC, hippocampus, and amygdala. For the mPFC OXTR mRNA level, the data revealed a significant difference among the groups (F_(4, 20)_ = 5.881, *p* = 0.003); further analysis exhibited that SPS-Veh group had lower mPFC OXTR mRNA level than the CON-Veh (*p* = 0.024) and SPS-OXT groups (*p* = 0.002), and ASB may further blocked the OXT effect (SPS-ASB/OXT group comparing to SPS-OXT group, *p* = 0.055) (Fig. 5a). For the hippocampal OXTR mRNA level, one-way ANOVA exhibited a significant difference among all groups (F_(4, 20)_ = 6.098, *p* = 0.002), deriving from the lower hippocampus OXTR mRNA level in SPS-Veh (*p* = 0.024), CON-OXT (*p* = 0.007), and SPS-OXT groups (*p* = 0.003) compared to the CON-Veh group (Fig. 5b). For the amygdala OXTR mRNA level, ANOVA revealed a significant difference among the groups (F_(4, 20)_ = 8.060, *p* < 0.001); further analysis exhibited that the hippocampus OXTR mRNA level was lower in SPS-Veh (*p* = 0.001), CON-OXT (*p* = 0.022), SPS-OXT (*p* < 0.001), and SPS-ASB/OXT groups (*p* = 0.014) than the CON-Veh group (Fig. 5c).

For the mPFC OXTR protein expression, ANOVA revealed a significant difference among the groups (F_(4, 15)_ = 9.21, *p* < 0.001); further analysis exhibited that the SPS-Veh rats had lower mPFC OXTR protein expression than the CON-Veh (*p* = 0.023) and CON-OXT (*p* = 0.002). Besides, SPS-ASB/OXT rats also showed lower mPFC OXTR protein expression than the CON-Veh (*p* = 0.03) and CON-OXT (*p* = 0.002). In addition, SPS-OXT showed a trend of elevated the mPFC OXTR protein expression compared with the SPS-Veh (*p* = 0.053) and SPS-ASB/OXT (*p* = 0.069) (Fig. 5d). For the hippocampal OXTR protein expression, there was no significant difference among the groups (F_(4, 15)_ = 0.62, *p* = 0.655) (Fig. 5e). For the amygdala OXTR protein expression, the data revealed a significant difference among the groups (F_(4, 15)_ = 14.005, *p* < 0.001); further analysis exhibited that the SPS-Veh rats had lower amygdala OXTR protein expression than the CON-Veh (*p* = 0.03), CON-OXT (*p* = 0.005), and SPS-OXT rats (*p* = 0.02). Moreover, SPS-ASB/OXT group also showed lower amygdala OXTR protein expression than the CON-Veh (*p* = 0.001), CON-OXT (*p* < 0.001), and SPS-OXT groups (*p* < 0.001) (Fig. 5f).

**Fig. 6:**
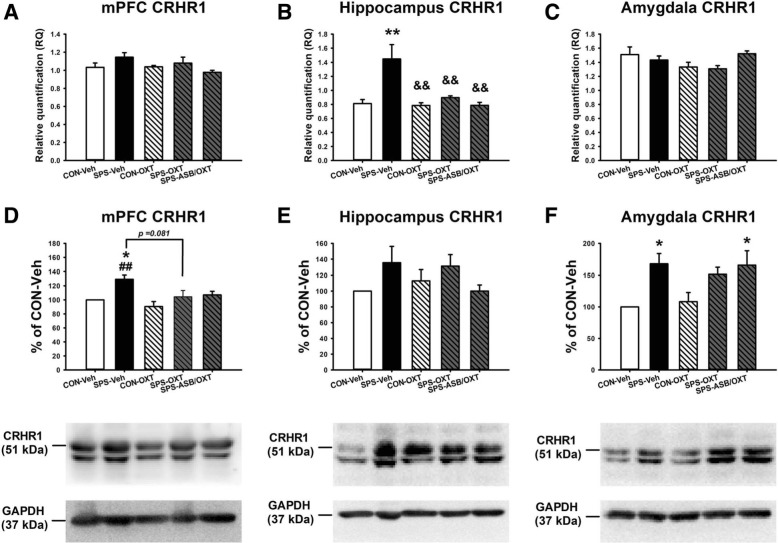
The CRHR1 mRNA levels and protein expressions in the mPFC, hippocampus, and amygdala. OXT reversed the SPS-elevated the hippocampus CRHR1 mRNA level, but SPS and drugs (OXT and ASB) did not affect the CRHR1 mRNA level in mPFC and amygdala (**a**-**c**). SPS increased the CRHR1 protein expressions in the mPFC and amygdala, but only the mPFC CRHR1 protein expressions could reverse by OXT (**d**, **f**). SPS and drugs (OXT and ASB) did not affect the hippocampus CRHR1 protein expression (E). SPS = single prolonged stress, VEH = vehicle, OXT = oxytocin, ASB = atosiban, OXTR = oxytocin receptor. Bars represent mean ± SEM; *p < 0.05, **p < 0.01, compared with Control-Veh; ##*p* < 0.01, compared with Control-OXT; ^&&^*p* < 0.01, compared with SPS-OXT. N = 5 for the mRNA levels (**a**-**c**); N = 4 for the protein expressions (**d**-**f**)

For the mPFC CRHR1 mRNA level, the data revealed no significant difference among the groups (F_(4, 20)_ = 1.966, *p* = 0.139) (Fig. 6a). For the hippocampal CRHR1 mRNA level, one-way ANOVA exhibited a significant difference among all groups (F_(4, 20)_ = 8.435, *p* < 0.001), and this difference was derived from the higher hippocampus CRHR1 mRNA level in SPS-Veh group compared with CON-Veh (*p* = 0.002), CON-OXT (*p* = 0.001), SPS-OXT (*p* = 0.006) and SPS-ASB/OXT groups (*p* = 0.001) (Fig. 6b). For the amygdala CRHR1 mRNA level, one-way ANOVA revealed no significant difference among the groups (F_(4, 20)_ = 2.038, *p* = 0.128) (Fig. 6c).

For the mPFC CRHR1 protein expression, ANOVA revealed a significant difference among the groups (F_(4, 15)_ = 5.277, *p* = 0.007); further analysis exhibited that the SPS-Veh rats had higher mPFC CRHR1 protein expression than the CON-Veh (*p* = 0.033) and CON-OXT (*p* = 0.004). Furthermore, OXT may reduce SPS-increased mPFC CRHR1 protein expression (*p* = 0.081) (Fig. 6d). For the hippocampal CRHR1 protein expression, there was no significant difference among the groups (F_(4, 15)_ = 1.599, *p* = 0.226) (Fig. 6e). For the amygdala CRHR1 protein expression, the data revealed a significant difference among the groups (F_(4, 15)_ = 4.715, *p* = 0.012); further analysis exhibited that the SPS-Veh (*p* = 0.039) and SPS-ASB/OXT (*p* = 0.047) rats had higher amygdala CRHR1 protein expression than the CON-Veh (Fig. 6f).

**Fig. 7:**
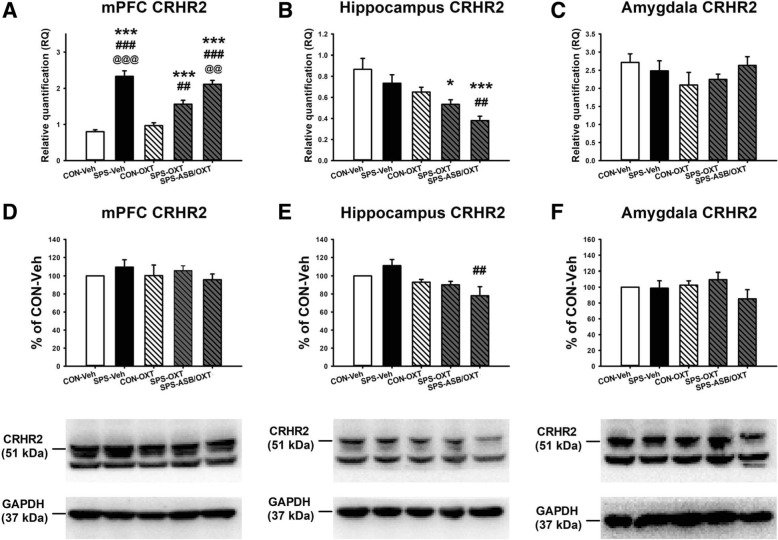
The CRHR2 mRNA levels and protein expressions in the mPFC, hippocampus, and amygdala. OXT could decrease SPS-increased mPFC CRHR2 mRNA level, and this effect could be blocked by ASB (**a**). OXT and ASB reduced the hippocampus CRHR2 mRNA level in SPS rats (**b**). SPS and drugs (OXT and ASB) did not affect the CRHR2 mRNA level in amygdala (C). SPS and drugs (OXT and ASB) did not affec the CRHR2 protein expressions in the mPFC, hippocampus, and amygdala, excepting ASB reduced hippocampus CRHR2 expression (D-F). SPS = single prolonged stress, VEH = vehicle, OXT = oxytocin, ASB = atosiban, OXTR = oxytocin receptor. Bars represent mean ± SEM; *p < 0.05, **p < 0.01, ***p < 0.01, compared with Control-Veh; ##*p* < 0.01, ###*p* < 0.001, compared with Control-OXT. N = 5 for the mRNA levels (A-C); N = 4 for the protein expressions (**d**-**f**)

For the mPFC CRHR2 mRNA level, ANOVA revealed a significant difference among the groups (F_(4, 25)_ = 41.712, *p* < 0.001); further analysis exhibited the SPS-Veh rats had higher mPFC CRHR2 mRNA level than the CON-Veh (*p* < 0.001), CON-OXT (*p* < 0.001), and SPS-OXT rats (*p* < 0.001). SPS-ASB/OXT rats also showed higher mPFC CRHR2 mRNA level than the CON-Veh (*p* < 0.001), CON-OXT (*p* < 0.001), and SPS-OXT rats (*p* = 0.008). Besides, SPS-OXT rats showed higher mPFC CRHR2 mRNA level than the CON-OXT rats (*p* = 0.004) (Fig. 7a). For the amygdala CRHR2 mRNA level, the data revealed a significant difference among the groups (F_(4, 25)_ = 7.517, *p* < 0.001), deriving from the difference between CON-Veh and SPS-OXT groups (*p* = 0.015); CON-Veh and SPS-ASB/OXT groups (*p* < 0.001); and SPS-Veh and SPS-ASB/OXT groups (*p* = 0.009) (Fig. 7b). For the amygdala CRHR2 mRNA level, one-way ANOVA revealed no significant difference among the groups (F_(4, 25)_ = 1.029, *p* = 0.412) (Fig. 7c).

For the mPFC CRHR2 protein expression, one-way ANOVA revealed no significant difference among the groups (F_(4, 15)_ = 0.524, *p* = 0.72) (Fig. 7d). For the hippocampus CRHR2 protein expression, ANOVA exhibited a significant difference among all groups (F_(4, 15)_ = 4.336, *p* = 0.016), this difference was derived from the difference between SPS-Veh and SPS-ASB/OXT groups (*p* = 0.009) (Fig. 7e). For the amygdala CRHR2 protein expression, there was no significant difference among the groups (F_(4, 15)_ = 1.164, *p* = 0.365) (Fig. 7f).

## Discussion

In the present study, we demonstrated that a rat model of SPS can be used to investigate the relationships among psychological trauma-impaired prosocial behavior and the profiles of OXT and CRH over fear circuit areas. Specifically, (i) SPS reduced rats’ inclination of staying at the sociable place with performing less prosocial contact. Intranasal OXT can amend this deficit in a pharmacologically specific manner, as the amending effect disappeared following the administration of OXT antagonist. (ii) These findings were greatly relevant to the changes of individuals’ willing of sociability, but not curiosity. (iii) OXTR became downregulated after SPS in mPFC and amygdala, the latter exhibited higher therapeutic specificity to intranasal OXT. (iv) Expression of CRHR1 appeared more sensitive than CRHR2 to SPS, where higher CRHR1 protein levels were found in mPFC and amygdala. Interpretations of these key findings are discussed below.

After traumatic stress, individuals are likely to react in a frightened manner and may avoid social interaction, which could be mediated by impaired OXT signaling [19, 21]. Our study demonstrated that OXT effectively assisted in restoring SPS-induced impairment of social behavior in rats. This was manifestly a specific OXT effect because it could be eliminated by blocking OXTR. Note the behavior of making a contact to the restrainer represents more inclination of executing prosocial behavior than a choice to enter zone 3, it is accordingly that the percentage of social contact time in zone 3 is an appropriate variable to examine the effects of SPS and OXT manipulations. Also note as the OXT effect did not happen if rats were not previously traumatized, indicating a stress-protective ability of OXT and in a way highlights the importance of social support to the one being psychologically distressed [2, 48].

OXT has been considered a stress buffering hormone to treat social interaction deficit in human [49] and to enhance social interaction in rats being previously socially defeated [50]. Our findings support this hypothesis by employing a different paradigm, i.e., rats showed less prosocial inclination towards the place where they were previously distressed, and that can also be restored by OXT. This is rather different with social defeat [50] as in our setting rats needs to deal with two different (and possibly in conflict with each other) situations, the one to show their sociability is similar to the one bearing fear memory. Taken together with our previous finding that OXT can mitigate SPS-Induced Impairment of fear extinction [22], the unforgettable fear should be highly responsible for the reduction of prosocial behavior. This phenomenon is less to do with the reduction of curiosity as there were no effects of SPS and OXT manipulation in our NORT experiment, which is in contrast to that novelty can lead to a change in social behavior in juvenile and non-distressed rats [51]. This discrepancy highlights that age and traumatic experience are crucial for individual to shift their attention between curiosity and social engagement. It is also possibly due to the inconsistent performance in novelty seeking of PTSD-like rats [38, 52, 53].

SPS not only affects individuals’ fear response and social behaviors, it also affects the receptors profile of implicated neurosubstrates. The present study demonstrated that following SPS, OXTR became downregulated in mRNA/protein levels in almost all three fear-associated areas (except hippocampal protein levels, which will be discussed later). This is along with the evidence that OXTR are highly sensitive to stress and in terms of both fear reaction [54, 55] and social behavior [56]. It is worth mentioning that in our study OXTR expression in the mPFC and amygdala reacted much stronger to OXT manipulations than that of hippocampus, exemplifying an area-dependent reactivity to stress, which was observed in CRHRs too. The mechanism underlying the incongruity of hippocampus from other fear circuit areas could be complicated, however it is possibly relevant to the unique characteristic of hippocampus, as suggested by standard model of system consolidation which highlights that hippocampus is responsible primarily in the initial stage of memory process, i.e., encoding, then the information moves to other places (normally the frontal cortex) in a more permanent form of storage [57]. In the present study, the most consistent data to unite behavioral and neurochemical findings was from the OXTR of mPFC, where SPS decreased both mRNA and protein levels of OXTR. The reduction of mPFC OXTR can be restored by OXT in a specific manner as it can be blocked by OXT antagonist ASB, indicating a diversity of central OXTR involvement in different brain regions [41], in which OXTR in the mPFC may regulate social behaviors [58]. Another study also supports our findings that interneurons with oxytocin receptor modulate social and emotional behaviors by acting on local medial prefrontal cortex (mPFC) circuits to coordinate responses to OXT and CRH [11].

One of the aims of the present study is to examine the role of CRH signaling in the OXT-mediated social behavior following psychological trauma, given that there are strong associations between OXT and HPA axis [27–29]. Our results demonstrated an area-dependent double dissociation of CRHRs, where the protein of CRHR1 appeared more affected by to SPS in mPFC and amygdala, mRNA of CRHR2 was found highly sensitive to SPS in mPFC only. This upregulation of CRHR2 mRNA level possibly refers to an anxiolytic effect mediated by CRHR2 [59] in buffering the SPS-induced conditioned anxiety [9]. The mechanism underlying the observation that expression of CRHR1 appeared more sensitive than CRHR2 to SPS can be complicated. However, it could be relevant to the evidences that CRHR1 exhibits higher affinity to CRH than CRHR2 [60] and the lack of CRHR1 leads to a greater influence to anxiety [61]. In terms of mPFC, a more interesting finding was that SPS rats exhibited lower OXTR but higher CRHR1 protein levels, which is along with the evidence that CRHR1 exerts some anxiogenic properties in rodents [62], whereas for OXT, anxiolytic [63]. In other words, It is possible that the reduced prosocial behavior observed in the present study can be to a degree due to the increase of anxiety toward the object where the animal being stressed.

Taken together, it appears that OXT and CRH are responsible differently to the trauma-induced psychological problems. The present study showed that central OXT is highly demanded in the prosocial choice of rats being previously distressed, CRH seems less involved in this mechanism. On the other hand, our SPS rats exhibited greater CRHR1 in mPFC together with a longer time staying in zone 2 (referring to anxiety/ambivalence, see [38, 64]) may have twofold implications. It is in line with the evidence that longer staying with ambivalence attenuates the neural circuit activity involved in controlling anxiety [65], and also in a way justifies the CRH regulation of anxiety. In addition, as the phenomenon was sensitive to OXT and OXT antagonist ASB (OXT shortened and ASB lengthened the time staying in zone 2), OXT signaling is also required. Taken together, our findings are along with the hypothesis that while OXT is primarily engaged in the performance of social behaviors, CRH is greatly involved in the regulation of stress and anxiety [11].

Several limitations/concerns of the present study should be addressed. Firstly, the mRNA and protein profiles were sometimes inconsistent. Possible explanations could be that changes in protein expression do not always reflect the changes in mRNA levels, possibly due to post-transcriptional modification [66] or because that proteins can be transported to other areas from its synthesized place [67]. Secondly, we are unable to eliminate the possibility that tested rats choosing the sociable place or made a social contact is entirely due to their prosocial idea (or possibly an empathetic approach), possibly it could be relevant to their curiosity toward the novel object too. As there were no effects of SPS and OXT manipulation in our NORT experiment, this confounding factor could be ruled out, i.e., the changes of prosocial behavior found in the present study should be less to relevant to novelty recognition. Thirdly, together with our previous finding that OXT is beneficial to restore fear memory extinction failure [22], the present study for a further step suggests that OXT may exert a therapeutic potential to remedy the impaired social behavior. However, we are unable to delineate whether these two psychological profiles are independent to each other. Finally, the method of local infusion of OXT is a more direct way to examine our hypothesis however it was not applied in our study thus our interpretation should be more cautious. Future study of central OXT effect is suggested to include the method of local infusion.

## Conclusions

Conclusively, the present study demonstrated that psychological trauma, such as SPS, may affect rats’ prosocial behaviors. We found that behaviorally OXT restored the SPS-impaired prosocial contacts, and neurochemically it reversed the SPS-reduced OXTR expressions in mPFC and amygdala. We confirmed that the trauma-impaired sociability is highly associated with OXT signaling pathway, whereas for the CRHRs, expression of CRHR1 appeared more sensitive than CRHR2 to SPS. The present study extends the clinical use of OXT by showing its therapeutic potential to amend the impaired social behavior of PTSD.

## Abbreviation

CORT: corticosterone
CRH: corticotropin-releasing hormone
CRHR1: corticotropin-releasing hormone receptor type 1
CRHR2: corticotropin-releasing hormone receptor type 2
NORT: novel object recognition test
OXT: oxytocin
OXTR: oxytocin receptors
PTSD: posttraumatic stress disorder
SCT: social choice test
SPS: single prolonged stress
